# Solitary Plasmacytoma of the Mandible: A Case Report

**DOI:** 10.30476/dentjods.2022.95247.1856

**Published:** 2023-03

**Authors:** Mahdie Zeinali, Nader Navabi, Mohammad Reza Zarei, Alireza Ranjbar Hassni

**Affiliations:** 1 Postgraduate, Dept. of Oral Medicine, Kerman Dental School, Kerman University of Medical Sciences, Kerman, Iran; 2 Dept. of Oral Medicine, Kerman Dental School, Kerman University of Medical Sciences, Kerman, Iran; 3 Student Research Committee, Dental School, Kerman University of Medical Sciences, Kerman, Iran

**Keywords:** Mandible, Neoplasms, Plasma Cell, *Plasmacytoma*

## Abstract

Plasmacytoma is an abnormal proliferation of monoclonal B-cells, and it can occur in several forms including multiple myeloma, solitary plasmacytoma of the bone, and extramedullary plasmacytoma primary. The solitary plasmacytoma of the bone accounts for about 3-10% of all plasma cell neoplasms, and occurs most often in the vertebrae, whereas the solitary plasmacytoma of the mandible is an extremely rare occurrence. This case report presents a 61-year-old woman with various underlying diseases diagnosed with solitary plasmacytoma of the mandible. This case is very well documented with radiographic imagery, clinical, and histopathological findings.

## Introduction

Plasma cell neoplasm or plasmacytoma is an aberrant proliferation of monoclonal B-cells with a prevalence of between 3.3% and 2.6% per 100,000 people [ 1 - 4 ]. Plasmacytoma arises in three distinct types including (1) multiple myeloma (MM), a cancer of the bone marrow with a deadly and destructive nature that is the most prevalent plasma cell neoplasm, affects multiple bones in osteolytic foci with abundant plasma cells; ( 2) solitary plasmacytoma of bone (SPB), which develops in soft tissue or the maxillary sinus, and (3) extramedullary primary plasmacytoma (PEMP), which develops in soft tissue or the maxillary sinus [ 1 ].

A total of 95 percent of SPB types are most common in adults over the age of 40, with men having a higher prevalence [ 5 ].SPB is uncommon in the jaw, accounting for only 4% of all occurrences of plasmacytoma in this region. SPB in the jaw has a poor prognosis; with 50% of cases progressing to MM [ 5 - 6 ].This case report pertains to an older patient who was diagnosed with SPB.

## Case Presentation

A general dentist referred a 61-year-old woman to the Department of Oral Medicine and Orofacial Pain at the School of Dentistry of Kerman University of Medical Sciences due to pain and swelling in the right mandibular gum that had begun around two months before. A systemic evaluation indicated that she had a history of diabetes mellitus, hypercholesterolemia, hypertension, and hypothyroidism. She reported daily use of metformin, glibenclamide, losartan, metoprolol, atorvastatin, and levothyroxine.

Initial clinical examination indicated a mild, antero-posteriorly extending enlargement on the right mandibular body that was painless on palpation, covered with normal, intact mucosa and had a hard, bony consistency.

**Figure 1:**
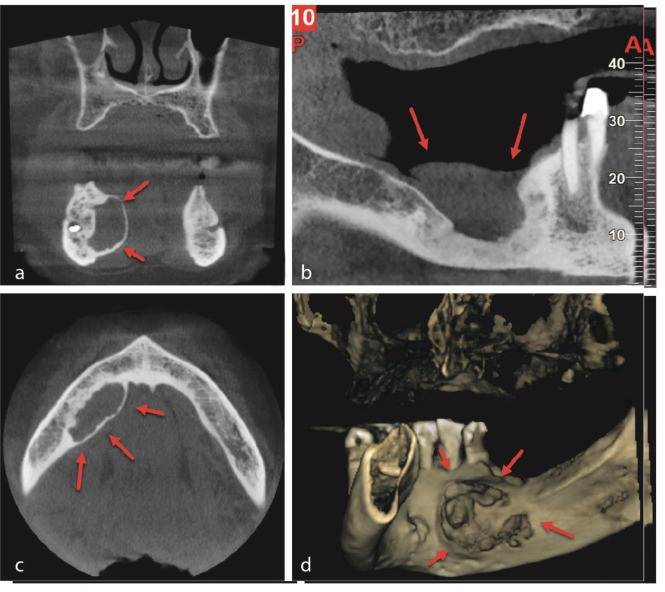
Cone beam computed tomography (CBCT) scans depicting the lesion at various views (Red Arrow); **a:** Coronal View, **b:** Sagittal View, **c:** Axial View, **d:** Full 3D View

On panoramic radiography and cone-beam computed tomography (CBCT) scanning, a single elliptical unilocular mixed radiolucent-radiopaque lesion in the right body of the jaw (3.5cm in length and 2.5cm in height) without a sclerotic rim was observed. The lesion extended antero-posteriorly from the mesial aspect of the right mandibular canine to the right first mandibular region (the patient had only eight natural teeth in the anterior segment of the mandible initially), but no extension was noticed to the alveolar crest and lower border of the mandible. Additionally, the lesion did not result in any pathologic alterations to the adjacent anatomical structures, such as root resorption or displacement. The vital adjacent teeth were non-sensitive to percussion. The aspiration produced a negative result. The lesion expanded and eroded the lingual cortex (Figure 1).

The results of all laboratory tests, including a complete CBC, ESR, CRP, calcium, blood urea nitrogen, creatinine, albumin, and immunoglobulin serum electrophoresis were normal.

The suggested differential diagnosis for this intraosseous lesion included ameloblastoma, osteosarcoma, mucoepidermoid carcinoma, lymphoma, simple bone cyst, central giant cell granuloma, cemento-osseous dysplasia, keratocyst odontogenic tumor, metastatic malignant lesion, abnormalities of the blood vessels, and residual cysts.

**Figure 2:**
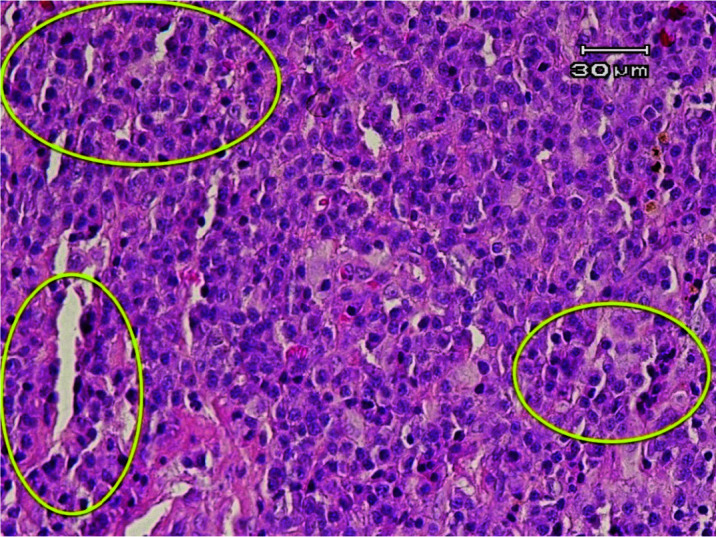
Histopathological view depicting sheets of plasma cells (Within Yellow Circles)

Under local anesthetic and sterile settings, an intraosseous incisional specimen, consisting of the premolar tooth and various pieces of soft and hard cream-brown tissue measuring 1*1*0.4 cm in total, was obtained through a complete mucoperiosteal flap. Multiple plasma cell sheets were observed in the primary sample (Figure 2). Immunohistochemistry staining for CD138 showed a positive reaction in plasmacytoid cells, which confirmed the plasmacytoma diagnosis. The second procedure was an excision, followed by a bone marrow aspiration and a biopsy to rule out multiple myeloma.

**Figure 3:**
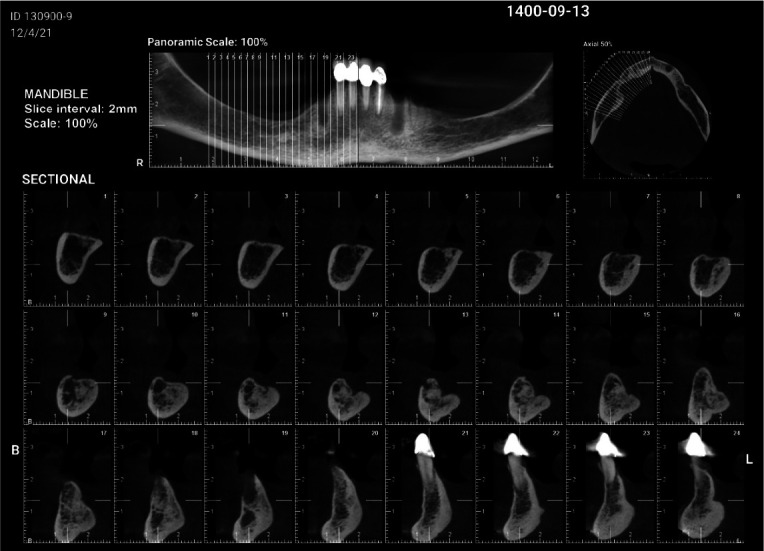
2-year cone beam computed tomography (CBCT) follow-up shows healing at the site of the lesion

The findings indicated cellular marrow with 7% plasma cells. An abdominal ultrasound of the liver, bile ducts, spleen, pancreas, and kidneys was ordered to examine other potentially affected foci. However, all organs were found to be normal, and no involvement was suspected. The test for urinary monoclonal protein was negative as well. After being diagnosed with SPB, the patient was referred to an oncologist and received a total of 5000 cGy of radiation over the course of 25 radiotherapy sessions during 35 days. No clinical signs of recurrence of the lesion were seen over the 2-year follow-up period. Bone healing was observed in surgery and radiation sites when CBCT imaging was performed (Figure 3).

Bone formation progressed from the periphery to the center, but healing in the surgical defect's central regions had not yet been completed. A PET scan was requested for the patient to evaluate the development of new lesion foci, which showed no new lesion foci in the body.

## Discussion

An SPB incidence in the jawbones is a rare occurrence. The mandible is involved far more commonly than the maxilla. The angle, ramus, and molar are the most affected mandibular regions [ 5 , 6 ].SPB also occurred in the posterior jaw of the current patient, and it appears that the posterior mandible and its ramus had a higher rate of involvement due to the presence of more bone marrow spaces. In their analysis of documented instances of SPB, Kanazawa *et al*. [ 4 ] found that the incidence of this lesion is higher in men and that the onset age spans from 34 to 76 years. The median age of patients is 53, nearly ten years younger than the median age of individuals with multiple myeloma. The age and gender of the present patient, a 61-year-old female, did not match those reported by Kanazawa *et al*. [ 4 ]. Nonetheless, two SPB cases reported by Kucukkurt *et al*. [ 7 ] share some similarities with the current case. The instances involved a 67-year-old woman and a 61-year-old woman with SPB in the mandible; both patients complained of pain, and the site of the lesion in both cases was the ramus. Although the cause of SPB is unknown, persistent inflammation, radiation, viral infections, and genetic alterations have been linked to its development [ 1 ]. Regarding chronic irritation, Poggio [ 8 ] reported a case where the titanium surface of a dental implant may have triggered B-cell growth in the mandibular bone space. The aforementioned patient in this case had multiple systemic diseases, including diabetes and hypertension. Caliskan *et al*. [ 9 ] and Yoon *et al*. [ 10 ] found SPB in the jaws of two patients who had had kidney transplantation. However, establishing an association between these two disorders requires additional data.

SPB is characterized by nonspecific symptoms, rendering clinical diagnosis challenging. The most prevalent form of SPB is characterized by a persistent swelling of the jaw accompanied by pain, paresthesia, pathologic bone fractures, and loose teeth [ 1 , 4 , 6 ]. Additionally, the present patient's symptoms appeared as gingival soreness and swelling. However, Kanazawa *et al*. [ 4 ] have determined that the lesion is more likely to be painless.

SPB should be diagnosed based on clinical signs and symptoms, radiographic manifestations, hematological and histological tests, and the absence of systemic alterations related to MM (such as hypercalcemia and anemia with renal involvement) [ 1 ].

Despite the fact that the present patient's radiological signs were a solitary, unilocular radiolucency without a sclerotic rim, incisional surgery and the finding of histological alterations consistent with SPB led to a conclusive diagnosis. SPB diagnosis by radiography alone can be difficult at times. Shah and Sarkar observed a plasmacytoma of the jaw that resembled a large periapical cyst radiographically [ 11 ]. Liu and Li emphasized the relevance of CT scans in such patients and indicated that the histological symptoms of SPB lesions were consistent with CT scan results in the SPB cases they analyzed [ 12 ].

This patient was treated with a combination of excisional surgery and radiation therapy. SPB has a more favorable outlook than MM. Rullo *et al*. [ 1 ] reported a 5-year SPB survival rate of 60% [ 1 ]. Due to the likelihood of SPB transforming into MM over time, patients with this lesion should be closely monitored. Two years following the conclusion of radiation, neither clinical nor radiological indicators of recurrence of the lesion were detected in the current patient. Given that the average survival rate for MM is just four years, the disease is essentially incurable and the treatments given to patients are palliative [ 2 ], the relevance of SPB case follow-up becomes apparent. The suggested radiation regimen for SPB is 50–40 cGy, which can achieve local control of the lesion of up to 80% [ 2 ]. The patient's radiation dose fell within the upper limits of the recommended dose range.

During a 2-year follow-up, neither recurrence nor conversion to MM was found in this patient. Kanazawa *et al*. [ 4 ] stated that there is no assurance regarding the association between SPB and MM. The patient with SPB reported by Matsumura *et al*. [ 5 ] was also symptom-free throughout their 1-year follow-up, but they indicated that additional follow-ups are required to confirm the absence of MM in these individuals. In their examination of records of SBP of the jaws, Agostini *et al*. [ 6 ] claimed that only 50 cases of SP of the jaw had been documented over a period of about 60 years (1948 –2011), and in half of these cases, SP progressed into MM, worsening the prognosis. The authors affirm that they have obtained all necessary consent forms from patients.

## Conclusion

SPB is rare in the mandible. In a 2-year follow-up, there were no indications of malignancy in the described case. The diagnosis of mandibular or maxillary SPB necessitates long-term patient monitoring. Moreover, in the differential diagnosis of radionuclides of the mandible, they should always consider unusual entities such as SPB. There is very little information in the literature regarding the CBCT findings of SPB, and one of the objectives of this case report was to characterize such findings. On CBCT, a solitary, elliptical, unilocular, well-defined (but without a sclerotic rim), osteolytic mass which destroys bone cortex is suggestive of SPB.

## Conflict of Interest

The authors declare that they have no conflict of interest.
